# Odor processing in the cockroach antennal lobe—the network components

**DOI:** 10.1007/s00441-020-03387-3

**Published:** 2021-01-23

**Authors:** Debora Fuscà, Peter Kloppenburg

**Affiliations:** Biocenter, Institute for Zoology, and Cologne Excellence Cluster On Cellular Stress Responses in Aging-Associated Diseases (CECAD), University of Cologne, Zülpicher Str. 47b, 50674 Cologne, Germany

**Keywords:** Olfaction, Antennal lobe, Projection neurons, Local interneurons, Periplaneta americana

## Abstract

Highly interconnected neural networks perform olfactory signal processing in the central nervous system. In insects, the first synaptic processing of the olfactory input from the antennae occurs in the antennal lobe, the functional equivalent of the olfactory bulb in vertebrates. Key components of the olfactory network in the antennal lobe are two main types of neurons: the local interneurons and the projection (output) neurons. Both neuron types have different physiological tasks during olfactory processing, which accordingly require specialized functional phenotypes. This review gives an overview of important cell type-specific functional properties of the different types of projection neurons and local interneurons in the antennal lobe of the cockroach *Periplaneta americana*, which is an experimental system that has elucidated many important biophysical and cellular bases of intrinsic physiological properties of these neurons.

## Introduction

In the previous decades, enormous progress has been made in understanding general mechanisms and principles of olfactory information processing at all levels of analysis - from the molecular and cellular to the circuit, system, and behavioral level. To a large extent, this progress was possible by taking advantage of comparative studies with various vertebrate and invertebrate experimental systems (see Laurent 2020). Overall, these studies showed very similar blueprints of olfactory systems not only between arthropods (mainly insects and crustacean) but also between arthropods and vertebrates (Hildebrand and Shepherd 1997; Strausfeld and Hildebrand 1999; Laurent et al. 2001; Wilson and Mainen 2006; Galizia and Rössler 2010; Harzsch and Krieger 2018). Pioneering studies on insects using behavioral, biochemical, morphological, and in vitro and in vivo physiological approaches have contributed significantly to this research from the very beginning. Among others, this included experiments on moths (Schneider 1969; Homberg et al. 1989), bees (Galizia and Menzel 2000), locusts (Laurent et al. 2001), and cockroaches (Boeckh et al. 1987). More recently, this insect zoo has been complemented by the fruit fly (Wilson 2013) and the red flour beetle (Dippel et al. 2016), thus adding molecular genetics to the repertoire of methods used to study the olfactory system of insects. In insects, the first synaptic processing of the olfactory input is performed in the antennal lobe (AL), which is considered the functional analog of the vertebrate olfactory bulb. The primary processing of olfactory information in the antennal lobe is performed by two key types of neurons: the local interneurons (LNs) and the projection (output) neurons (PNs). Both neuron types have different physiological tasks during olfactory processing, which accordingly require specialized functional phenotypes. While we briefly summarize the most important anatomical and functional aspects of the different processing stages from the antennae to the AL output neurons, the focus is on key cell type-specific functional properties of the different types of projection neurons and local interneurons in the AL of the cockroach *Periplaneta americana*, an experimental system that has elucidated many important biophysical and cellular bases of intrinsic physiological properties of these neurons.

## Functional organization of the antennae: sensilla and olfactory sensory neurons

Detection and processing of olfactory signals start at the main olfactory sense organ, the antenna. The cockroach antennae consist of a short scapus and a long flagellum with about 150 annuli, which are equipped with various, randomly distributed types of olfactory sensilla that house the olfactory sensory neurons (OSNs) (Schafer and Sanchez 1973; Schaller 1978). Based on morphological features, three main types of olfactory sensilla can be distinguished: perforated basiconic, trichoid, and grooved basiconic sensilla (Altner et al. 1977; Toh 1977; Schaller 1978; Watanabe et al. 2012), which correspond to the sensilla basiconica, trichodea, and coeloconica in the fruit fly and several moths (Keil 1999). For each of these main sensilla types, several subtypes have been described.

While the antennal OSNs of *P. americana* typically respond to various odorants, most OSNs can be physiologically categorized into groups with similar response spectra. OSN response spectra to single pure substances of different chemical classes are comprehensively reviewed by Watanabe et al. (2012). The response groups can, in turn, be correlated with the different morphological types of sensilla (perforated basiconic, grooved basiconic, and trichoid sensilla) (Schaller 1978; Sass 1978; Selzer 1984; Boeckh and Ernst 1987; Fujimura et al. 1991): (1) Perforated basiconic sensilla house two (in the short subtype) or four OSNs (in the long subtype) that are predominantly responsive to alcohols and terpenes, but also aromatic compound groups and esters. The long type houses two OSNs, which are exclusively sensitive to the primary compounds of the female sex pheromone in addition to two OSNs that are responsive to terpenes. (2) Grooved basiconic sensilla are divided into two different groups, a short subtype with physiologically defined OSNs and a long subtype with OSNs that have not yet been physiologically characterized. The short grooved basiconic sensilla contain either OSNs that are exclusively olfactory and respond to various chemical substances, including aldehydes, carboxylic acids, terpenes, and aromatic compounds, or they contain OSNs that respond to carboxylic acids together with thermosensory receptors. (3) In contrast to the relative odorant specific OSNs of basiconic sensilla (Boeckh and Ernst 1987; Fujimura et al. 1991), the OSNs of the trichoid sensilla have a much broader response spectrum and detect various chemical compounds. Interestingly, they are functionally organized in pairs. These OSN pairs respond antagonistically even to slow concentration changes of odorants and are referred to as ON and OFF OSNs (Schaller 1978; Hinterwirth et al. 2004; Tichy et al. 2005; Burgstaller and Tichy 2011; Hellwig et al. 2019).

The comprehensive studies on OSNs of trichoid sensilla from Tichy and co-workers and numerous studies on response properties of OSNs from other sensilla types (Sass 1978; Selzer 1984; Boeckh and Ernst 1987; Fujimura et al. 1991) have shown that the olfactory system of the cockroach uses the two parallel pathways from the basiconic and trichoid sensilla to encode the odorant identity and the moment-to-moment succession of odorant concentrations together with the rate at which concentration changes (reviewed in Tichy and Hellwig, 2018). Here, the activity of OSNs in the basiconic sensilla provides the information for the combinatorial representation of odorant identity, while the antagonistic responses of the ON and OFF OSNs in the trichoid sensilla provide information about concentration increments and decrements. Important here is that each ON and OFF OSN can adjust their gain for sensing odorant concentration and for sensing the rate of concentration changes to the dynamics of the odorant signal. When the rate of change decreases, both OSNs increase their sensitivity for the rate of change at the expense of the sensitivity for the instantaneous concentration. Together, this optimizes the antennal olfactory system to simultaneously identify odorants and detect even small and slow changes in odorant concentration, all of which are essential for accurate odor tracking.

The specific arrangement of OSNs on the antennae is a prerequisite to encode the spatial representation of odor detection on the antennae. Since the cockroach is a hemimetabolic insect, the basic structures of the brain are built up during embryogenesis and gradually increase in volume during postembryonic development. During every nymphal stage, new annuli with additional OSNs are added to the antennal flagellum proximal to the head (Schafer and Sanchez 1973; Watanabe et al. 2018). In turn, these OSNs form new layers of OSN terminals in the AL glomeruli from the core to the cortex. This age-specific and thus, segment-specific layer formation ultimately produces an antennotopic innervation of the glomeruli (Nishino et al. 2018; Paoli et al. 2020). The physiological relevance for spatial coding was confirmed for the macroglomerulus (MG) electrophysiologically (Hösl 1990; Nishino et al. 2018) and most recently by Ca^2+^ imaging (Paoli et al. 2020).

The OSNs, together with other sensory neurons, send their axons to the n-anterior or n-posterior antennal nerve (all orientations presented here are according to the neuraxis; terminology from studies referring to the body axis was converted accordingly, see Ito et al., 2014), depending on whether they originate from sensilla located on the n-anterior or n-posterior surface of the flagellum respectively (Nishino and Mizunami 2006, 2007). This topological segregation is known in other insects as well (for review, see Galizia and Rössler, 2010), however seemingly not of functional relevance.

**Fig. 1:**
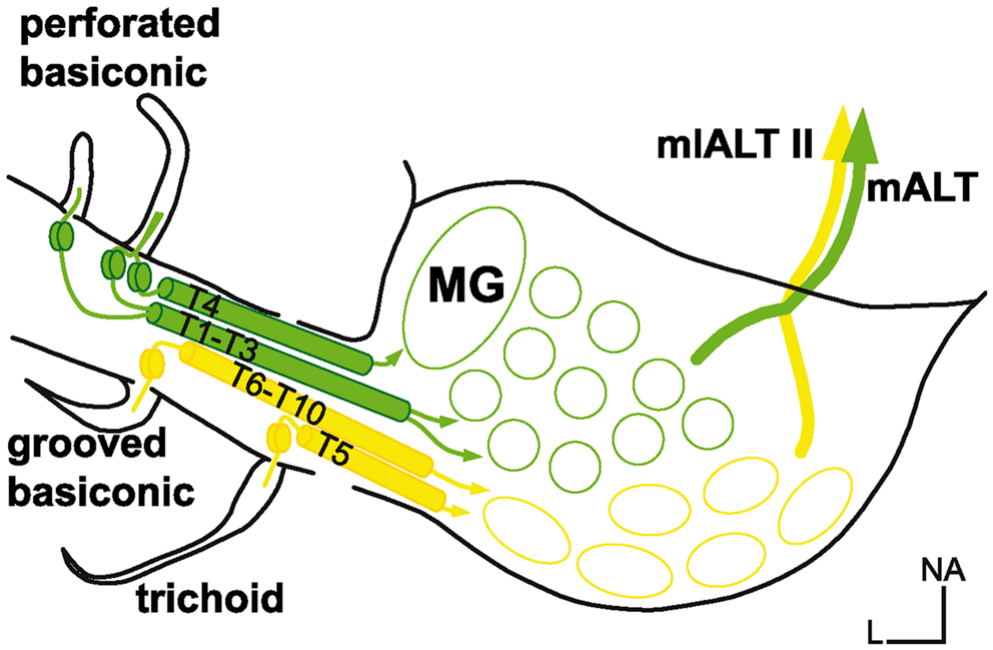
Schematic illustration of the pathways from the periphery to higher brain centers in the protocerebrum. OSNs of perforated basiconic sensilla (green) run through antennal tracts T1-T4 and terminate in the n-anteroventral group of glomeruli. PNs with dendrites in these glomeruli project through the mALT to the protocerebrum. OSNs in trichoid and grooved basiconic sensilla (yellow) run through antennal tracts T5 and T6-10, respectively, and terminate in the n-posterodorsal group of glomeruli. PNs with dendrites in these glomeruli project through the mlALT II to the protocerebrum (Malun et al. 1993; Watanabe et al. 2012, 2017). L lateral, mALT medial antennal lobe tract, MG macroglomerulus, mlALT II mediolateral antennal lobe tract, NA n-anterior, T1-10 antennal tracts 1–10

Bundled in the antennal nerve, olfactory, hygrosensory, and thermosensory afferents project to specific glomeruli in the AL (Boeckh and Tolbert 1993; Nishikawa et al. 1995; Nishino and Mizunami 2006, 2007; Nishino et al. 2010). The OSN axons collate into ten different antennal tracts that project into the AL with a sensillum specific topography (Watanabe et al. 2012): OSNs from perforated basiconic sensilla project through tracts 1–4 that terminate in glomeruli of the n-anteroventral region of the AL. OSNs from trichoid sensilla project through tracts 5–8, of which more than 90% run through tract 5 that terminates in a group of n-posteroventrally located glomeruli. At the same time, the remaining few OSNs follow tracts 6–8 to glomeruli in the n-dorsal AL region. OSNs from grooved basiconic sensilla project through tracts 6–10 terminating in glomeruli of the n-dorsal region of the AL (Fig. 1). Functionally, no explicit role has been assigned to the different tracts yet. However, the PNs that make up some of these tracts are well characterized so that certain conclusions can be drawn about these tracts' function (see below).

## Antennal lobe: basic circuit structure

In the central nervous system, complex neural circuits have evolved to process olfactory information efficiently. The primary olfactory input is intensively processed by a highly interconnected network of different types of PNs and LNs before the PNs transmit the processed information into higher-order processing regions of the protocerebrum. The basic structure of these circuits is very similar across phyla and usually follows a general scheme with variations in the numbers of its neuropilar subunits, the glomeruli, and cellular components (Strausfeld and Hildebrand 1999; Galizia and Rössler 2010). In this way, the neuronal circuitry in the olfactory system of *P. americana* follows the typical (insect) blueprint. In insects, the first synaptic processing station for olfactory information is the AL, which is the functional analog of the vertebrate olfactory bulb. The antennal OSNs, typically each expressing a single functional receptor gene, send their axons to the AL, where they collate by receptor type and converge into specific glomeruli. In the glomeruli, they form synapses with PNs and LNs. The PNs relay information to higher-order neuropils. The LNs mediate complex inhibitory and excitatory interactions between and within the glomeruli to form the olfactory representation in the AL, which ultimately shapes the tuning profile of PNs.

On the macroscopic level, the cockroach AL is compartmentalized into ~ 205 identifiable glomeruli, segregated into an n-anteroventral group of small oval glomeruli, including two sexually dimorphic glomeruli that are fused into the MG of the male, and an n-posterodorsal group of large, uniquely shaped glomeruli (Salecker and Boeckh 1996; Nishino and Mizunami 2006; Watanabe et al. 2010). The axons of olfactory, hygro-, and thermosensory neurons each project into a single glomerulus (Watanabe et al. 2010, 2012). In this process, they follow a stereotypical projection pattern, which leads to a sensillum type-specific organization of distinct glomerular clusters.

Most somata of the olfactory AL neurons are located in the ventrolateral somata group (VSG), where the somata of the different neuron types are organized in well separated and identifiable clusters. The vast majority of the cell bodies in the ventral part of the VSG belong to a cluster of uniglomerular PNs (uPNs). Somata of different sizes, which are located in the dorsal part of the VSG, usually belong to LNs. Directly dorsal to the uPN somata is a relatively homogenous, densely packed soma group belonging to spiking type I LNs, whose neurites give rise to the Y-shaped tract and exhibit GABA-like immunoreactivity (Distler 1989; Distler and Boeckh 1997a; Husch et al. 2009a). The somata farther dorsally belong mostly to a group of nonspiking LNs that are referred to as type IIa and type IIb LNs (Husch et al. 2009a, b).

## Synaptic connectivity within the Glomeruli

Within the glomeruli, the OSNs form synapses with the second-order neurons. Among them are several types of LNs, whose ramifications are confined to the AL and which mediate inter- and intraglomerular signal transmission, and uni- and multiglomerular projection neurons (uPNs and mPNs), which project from the AL to the protocerebrum. In *P. americana,* the ultrastructural organization of the glomerular neuropil has been analyzed in detail by early electron-microscopic studies, in which combinations of various labeling techniques were used to identify synaptic connections between different neuron types. These studies, which focused on OSNs, GABA-lir LNs, and uPNs, demonstrated synaptic connections between almost any possible combinations of these neuron types (Malun 1991a, b; Distler and Boeckh 1996, 1997b, a; Distler et al. 1998): OSNs form dyadic output synapses onto a uPN and a GABA-lir LN or mPN or onto two GABA-lir processes. Serial synaptic connections between OSNs and uPNs have been identified via a single interposed GABA-lir process or two interposed GABA-lir processes, creating both an inhibitory and a “disinhibitory” circuit motif. GABA-positive neurons form dyadic output synapses onto a uPN and an OSN or another GABA-lir process. Additionally, reciprocal synapses are present between uPNs and GABA-positive neurons, as well as between two GABA-positive processes. Input synapses from uPNs onto OSNs have not been found.

Paired recordings confirmed the functionality of chemical synapses between cholinergic uPNs and GABAergic LNs, and between different GABAergic LNs (Warren and Kloppenburg 2014). uPNs form strong excitatory synaptic connections to LNs and other uPNs mediating fast synaptic transmission via acethylcholine receptors. Synaptic input that was induced by spike trains in presynaptic LNs elicited inhibitory postsynaptic potentials (IPSPs) in both uPNs and LNs. The IPSPs were composed of both slow, sustained components and fast, transient components, which were coincident with presynaptic action potentials. The fast IPSPs were mediated by the GABA_A_ receptors, whereas the GABA_B_ receptors mediated the slow, sustained IPSPs.

## Component neurons of the antennal lobe network

### Projection neurons

**Fig. 2:**
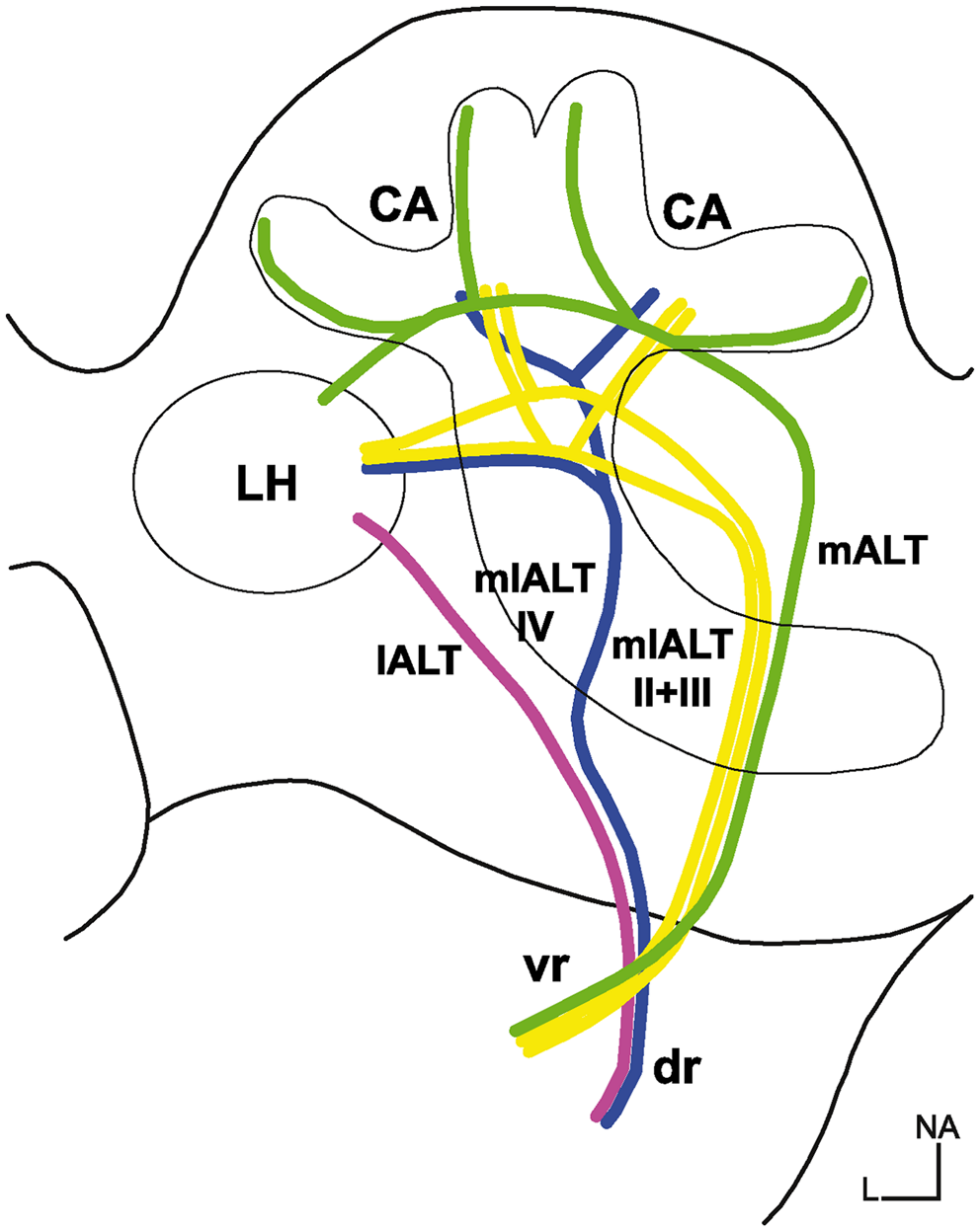
Schematic drawing of the five antennal lobe tracts that connect the antennal lobe and the protocerebrum. Courses of the tracts after (Malun et al. 1993). CA calyx, dr dorsal root, L lateral, lALT lateral antennal lobe tracts, LH lateral horn, mALT medial antennal lobe tract, mlALT II-IV mediolateral antennal lobe tract II-IV, vr ventral root

**Fig. 3:**
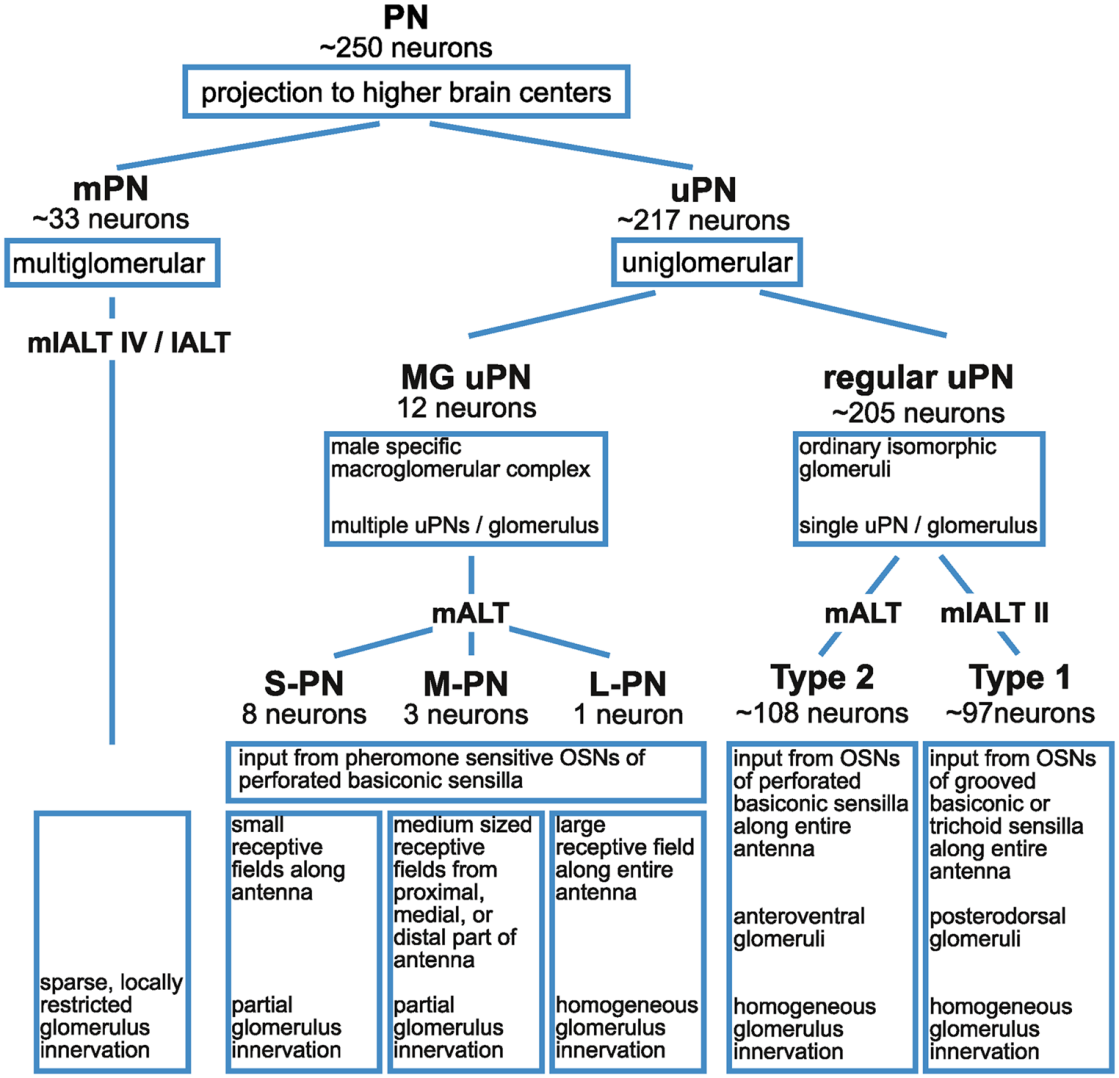
Inventory of antennal lobe projection neurons (PNs). Properties were described in (Malun et al. 1993; Strausfeld and Li 1999a; Watanabe et al. 2012, 2017; Nishino et al. 2018). Numbers are estimated as follows: ~ 250 PN axons were counted in different antennal lobe tracts (Boeckh et al. 1984; Malun et al. 1993). Watanabe et al. (2010) counted ~ 205 glomeruli in the antennal lobe of *P. americana*, ~ 108 n-anteroventral glomeruli, which are innervated by type 2 uPNs, and ~ 97 n-posterodorsal glomeruli which are innervated by type 1 PNs. According to the one uPN per glomerulus rule, these numbers correspond to the numbers of regular uPNs. Together with the 12 uPNs innervating the macroglomerular complex (Nishino et al. 2018), it adds up to ~ 217 uPNs, which leaves ~ 33 neurons with axons in antennal lobe tracts that consequently belong to mPNs. (l/m)ALT, (lateral/medial) antennal lobe tract; MG, macroglomerulus; (m/u)PN, (multiglomerular/uniglomerular) projection neuron; (L/M/S)-PN, (large-/medium-/small-sized) receptive field macroglomerular projection neuron

PNs constitute the main output pathway from the AL to the protocerebrum. Together with the OSNs they form a monosynaptic pathway from the antennae directly to the protocerebrum. Furthermore, they can receive synaptic input from and deliver synaptic input to LNs (Distler and Boeckh 1997b, a; Warren and Kloppenburg 2014), suggesting that they also contribute to information processing within the AL. There are essentially two main types of projection neurons: uPNs, which receive synaptic input exclusively in one glomerulus, and mPNs, which integrate synaptic input from many glomeruli. Both PN classes project into the protocerebrum via 5 distinct antennal lobe tracts (ALT; Fig. 2). These tracts were formerly termed antenno-cerebral tracts (Malun et al. 1993). Following the unified nomenclature of Ito et al. (2014), they are now called medial ALT (mALT), mediolateral ALT II-IV (mlALT II-IV), and lateral ALT (lALT). The 5 tracts arise from two common roots in the deutocerebrum. While uPN axons are bundled in tracts of the n-ventral root, mPN axons all run through tracts of the n-dorsal root (Malun et al. 1993). The n-ventral root gives rise to the medial ALT (mALT, former inner antenno-cerebral tract, iACT) and mlALT II and III, while the n-dorsal root gives rise to the mlALT IV and lateral ALT (lALT, former outer antenno-cerebral tract, oACT). The main PN types and their features are summarized in Fig. 3. In this context, it should be mentioned that uPNs are relatively well characterized while the data about mPNs are sparse.

### Uniglomerular projection neurons

The best characterized AL output neurons are the uPNs. Each uPN obtains synaptic input in a single glomerulus and sends its axon from the AL to the mushroom body calyces and the lateral horn (LH) of the protocerebrum. They are cholinergic and thus provide excitatory input to their postsynaptic targets in the protocerebrum, and in the input glomerulus of the uPNs also to LNs (Fusca et al. 2013; Warren and Kloppenburg 2014). While each of the sexually isomorphic (ordinary) glomeruli typically gives rise to only one uPN, the pheromone sensitive MG of the male gives rise to several uPNs.

#### uPN subgroups that arise from the ordinary glomeruli

While uPNs appear to constitute a functionally homogeneous neuronal population, they can be distinguished and classified by their respective input glomerulus and target regions. Two subtypes of uPNs innervating ordinary glomeruli can be differentiated, that have somata in two separate groups within the uPN cluster of the VSG, and receive input in different groups of glomeruli and project into distinct zones of the calyces (Figs. 1, 2, 3) (Malun et al., 1993; Strausfeld and Li, 1999a, b; Watanabe et al., 2017). Type 2 uPNs have their neurites in the n-anteroventral group of small oval glomeruli that receive input from perforated basiconic sensilla. Their mushroom body terminals are concentrated at the rim of the calyces (zone I Strausfeld and Li, 1999a, b) and the anterior part of the LH. These neurons have their somata in the n-anterior group of the uPN cluster and correspond to the mALT (= iACT) neurons, according to Malun et al. (1993). Because type 2 uPNs receive input from OSNs of the perforated basiconic sensilla (Watanabe et al. 2012, 2017), it is likely that the mALT carries predominantly information about odorant identidy. Type 1 uPNs on the other hand receive synaptic input in the n-posterodorsal group of large glomeruli, which are innervated by OSNs of the grooved basiconic and trichoid sensilla. Type 1 uPNs have their somata in the n-posterior group of the uPN cluster and project into the base and mid regions of the calyces (zones III, IIIA, Strausfeld and Li, 1999a, b) and the central region of the LH. These neurons likely correspond to the mlALT II (= ACT II) neurons described by Malun et al. (1993). Those neurons were characterized by an axon running through the mlALT II, a tract that runs nearby the mALT and can be considered a side branch of that tract. Like type 1 uPNs, mlALT II neurons were described to have somata located in a separate cluster posterior to the type 2 (mALT) uPN somata and were found to predominantly innervate the relatively large glomeruli in the dorsal region of the AL. Since type 1 uPNs obtain input from OSNs of the trichoid sensilla (Watanabe et al. 2012, 2017) it can be hypothesized that the mlALT II transfers primarily information about odorant concentration.

#### Macroglomerular uPNs

The male specific MG is functionally specialized and processes the primary component of the female sex pheromone periplanone-B. The somata of these uPNs are located within the cluster of type 2 uPNs, and they also project to the mushroom body calyces and the LH via the mALT. In contrast to ordinary glomeruli, which normally contain the neurites of only one uPN, the MG is innervated by multiple uPNs with spatially distinct receptive fields on the antenna (Hösl 1990; Nishino et al. 2018; Watanabe et al. 2018; Paoli et al. 2020). One PN, named “large receptive field” PN (L-PN) receives input from pheromone sensitive OSNs along the entire antennal flagellum and is hologlomerular. This neuron corresponds to the regular uPNs. In addition to the L-PN, the MG is occupied by several “medium-sized receptive field” PNs (M-PNs) and “small-sized receptive field” PNs (S-PNs) that cover specific sub-compartments of the glomerulus and receive input from medium-sized or small-sized receptive fields on the antenna, respectively. Note that pheromone-sensitive OSNs innervate the MG with an antennotopic organization; these specialized PNs, thus receive input from specific segments of the antenna only. Remarkably, with the sub-compartmentalized innervation of the MG by M-PNs and S-PNs, spatial information that is gained by the location of the OSNs on the antenna is preserved in the MG and conveyed to the protocerebrum.

#### Physiologal and biochemical properties of uPNs

PNs convey information over long distances. Accordingly, they generate (Na^+^-driven) action potentials upon odor stimulation or depolarizing current injection. Despite that uPNs receive primary sensory input in one glomerulus by one OSN type only, they usually respond to odorants of many different chemical classes with elaborate patterns that can include periods of excitation and inhibition (Husch et al. 2009a). This suggests that the responses are influenced by synaptic input of multiple origins. In this regard, it is interesting to note that type 2 uPNs usually have a very low frequency of spontaneous action potential or are even completely silent when not stimulated, while type 1 uPNs are often spontaneously active. (Strausfeld and Li, 1999a, b; Watanabe et al. 2017).

Immunohistochemistry, mass spectrometry, and functional pharmacology studies have shown that uPNs are cholinergic (Fusca et al. 2013; Warren and Kloppenburg 2014; Neupert et al. 2018) providing excitatory synaptic input to GABAergic type I LNs and neurons in the mushroom body and LH. In addition to the classical transmitter acetylcholine (ACh), they coexpress the peptide allatostatin-A (AST-A) (Neupert et al. 2018), but appear to lack the expression of multiple peptides as often observed in LNs (Fusca et al. 2015).

### Multiglomerular projection neurons

AL output is also conveyed by the multiglomerular (m)PNs via the n-dorsal root tracts (ALT IV and lALT). In contrast to the uPNs, the mPNs innervate many glomeruli, usually with only sparse, locally restricted arborizations in each glomerulus. Forming numerous axonal side branches, the mPNs project to the lateral protocerebrum and the LH, while the calyces are only sparsely innervated or not at all (Malun et al. 1993). The somata of the mPNs cannot be assigned to any distinct cell group but are scattered among somata of various sizes in the lateral deutocerebrum. Due to a lack of systematic electrophysiological studies (but see Strausfeld and Li (1999a, b), the function of these neurons remains enigmatic. As mPNs typically have neurites only in subglomerular portions and each glomerulus is covered by multiple mPNs, it is tempting to speculate that these neurons play a role in encoding spatial information similar to the S-PNs of the MG as discussed by (Galizia 2018; Paoli et al. 2020).

### Local interneurons

**Fig. 4:**
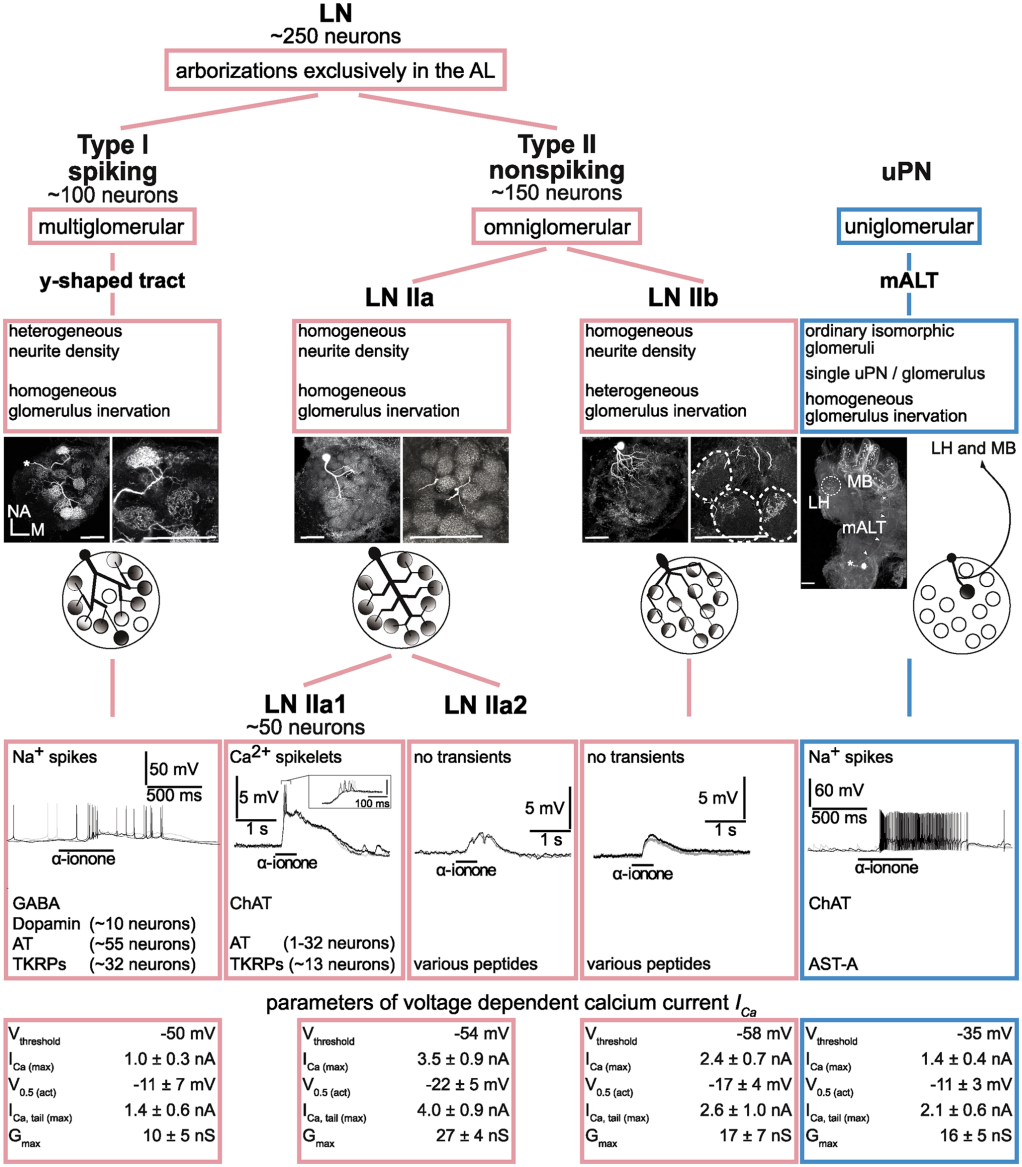
Inventory of the antennal lobe neurons with somata in the ventrolateral somata group (VSG). Data are summarized from the following sources: Morphological and physiological properties: (Ernst and Boeckh 1983; Boeckh et al. 1984; Husch et al. 2009a, b). Transmitter- and peptide content: (Neupert et al. 2012, 2018; Fusca et al. 2013, 2015). Odor responses: Overlays of three repetitive stimulations with the same odorant (Husch et al. 2009a, b). Numbers are estimated as follows: The VSG comprises ~ 500 neurons (Ernst and Boeckh 1983), of which ~ 250 belong to PNs with axons in different antennal lobe tracts (Boeckh et al. 1984; Malun et al. 1993). Accordingly, ~ 250 somata of the VSG belong to LNs. Fusca et al. (2015) counted ~ 300 ChAT-lir neurons that were grouped in several clusters and ~ 100 GABA-lir somata in the region were predominantly type I LNs are located. According to Husch et al. (2009a), all type I LNs are GABA-lir, and Distler (1989) stated that the great majority of GABA-lir somata in the VSG belong to LNs, while only a marginal portion of the axons in the antennal lobe tracts are GABA-lir. In summary, there are ~ 100 GABA-lir type I LNs, and if the majority of PNs (~ 250) are ChAT-lir, of the ~ 300 ChAT-lir somata ~ 50 belong to type IIa1 LNs (Fusca et al. 2013). AL antennal lobe, AST-A allatostatin-A, AT allatotropin, ChAT choline acetyltransferase, L lateral, LH lateral horn, LN I(II) type I(II) local interneuron, mALT medial antennal lobe tract, MB mushroom body, NA n-anterior, TKRPs tachykinin-related peptides, uPN uniglomerular projection neurons. Images: Orientation applies to all images. Positions of lacking somata are marked by asterisks. Scale bars, 100 µm. Original figures from Husch et al. (2009a, b)

In insects, local interneurons mediate complex excitatory and inhibitory synaptic interactions within and between the glomeruli (Christensen et al. 1998; Sachse and Galizia 2002; Chou et al. 2010; Huang et al. 2010; Yaksi and Wilson 2010; Fujiwara et al. 2014). Thereby, LNs help to structure the olfactory representation in the AL, ultimately shaping the tuning profile of the PNs. The LNs, whose synaptic input and output regions are restricted to the AL, constitute a heterogeneous population of neurons with distinct physiological, morphological, and biochemical properties (Seki and Kanzaki 2008; Chou et al. 2010; Seki et al. 2010; Tabuchi et al. 2015). Typically, they contain a classical transmitter like GABA or ACh and one or more potential co-transmitters like peptides or biogenic amines (Berg et al. 2007, 2009; Shang et al. 2007; Seki and Kanzaki 2008; Ignell et al. 2009; Kreissl et al. 2010; Chou et al. 2010). In *P. americana*, two main LN types with very different physiological properties can be differentiated (Fig. 4): (1) spiking type I LNs that generated Na^+^-driven action potentials upon odor stimulation and exhibited GABA-lir and (2) nonspiking type II LNs, in which odor stimulation evoked depolarizations, but no Na^+^-driven action potentials (Husch et al. 2009a). These marked physiological differences suggest important differences in their computational properties and synaptic output kinetics. It also implies that in the AL information is processed in parallel by LNs that use action potentials and LNs that use analog signals for intercellular communication.

#### Spiking type I local interneurons

Type I LNs generate Na^+^-driven action potentials upon odor stimulation or depolarizing current injection. They are broadly tuned and respond to different odorants with relatively similar firing patterns. Type I LNs give rise to the y-shaped tract (Distler 1990a; Husch et al. 2009a) and have a multiglomerular branching pattern with ramifications in many, but not all glomeruli, often including the MG. The density and pattern of ramifications vary between glomeruli from very dense to sparse, which could indicate a polarity with defined synaptic input and output regions.

#### Nonspiking type II local interneurons

In contrast to type I LNs, type II LNs do not generate Na^+^-driven action potentials. Odor stimuli induce graded changes of different complexity in the membrane potential. Type II LNs are omniglomerular, with branches in all glomeruli, including MG. The density and pattern of arborizations are similar in all glomeruli of a given type II LN but vary between different type II LNs (Husch et al. 2009a). Based on morphological and functional features, type II LNs constitute two subcategories, type IIa and type IIb LNs (Husch et al. 2009b).

In type IIa LNs, the ramifications are similar and evenly distributed over each glomerulus of an individual neuron. In type IIb neurons, on the other hand, the branches cover only parts of each glomerulus, often in a specific layer (Husch et al. 2009b). Whereas both type IIa and IIb LNs are broadly tuned, they also differ in their electrophysiological responses to olfactory stimuli. While odor responses in type IIb LNs are characterized by sustained, relatively smooth depolarizations, the responses of type IIa LNs are typically more complex, showing odor-specific elaborate patterns of excitation that can include Ca^2+^-driven spikelets in a subset of type IIa LNs, or hyperpolarization.

#### LN type–specific intrinsic properties

**Fig. 5:**
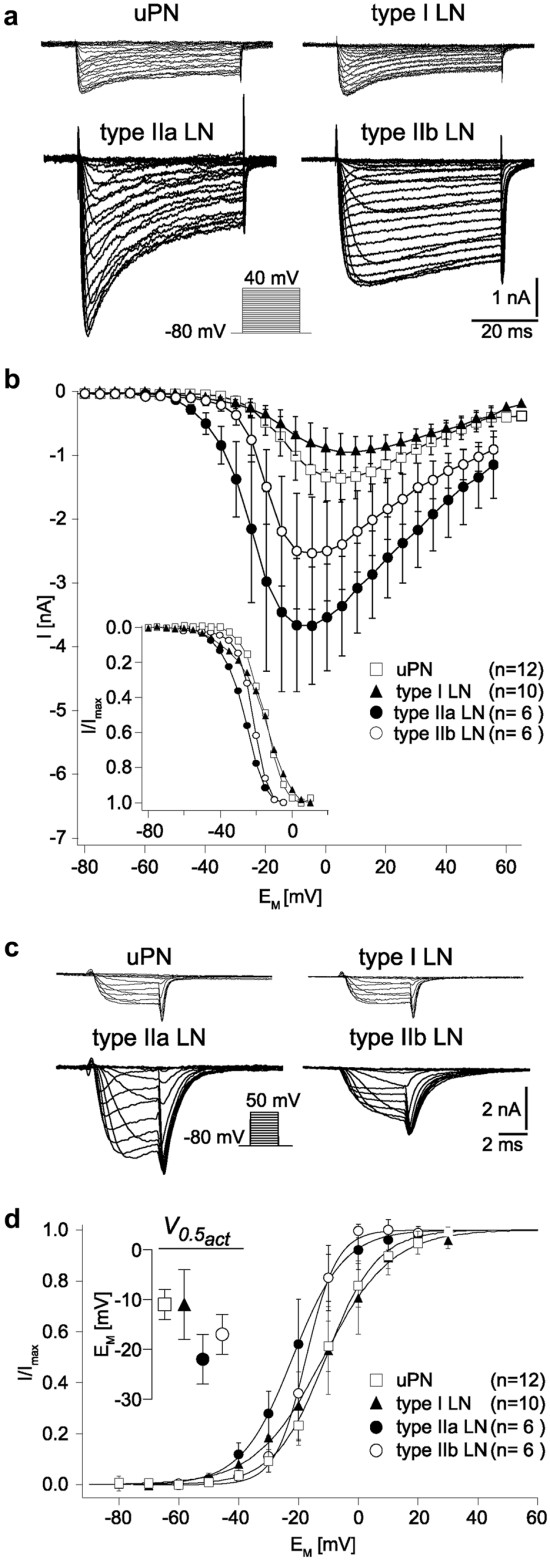
Ca^2+^ currents (*I*_*Ca*_) from uPNs, type I LNs and type II LNs. **a**, **b** Steady-state activation of *I*_*Ca*_ in uPNs, type I LNs, and type IIa and IIb LNs. Original traces (**a**) and current–voltage relations (**b**). The inset shows the relative current amplitudes, normalized to the maximum current of each cell type. **c**, **d** Tail currents of *I*_Ca_ (*I*_Ca, tai*l*_). Original traces (**c**) and current-voltage relations of normalized tail currents (**d**). Before averaging, the tail currents were normalized to the maximum tail current amplitude of each cell. Curves were fit to a first-order Boltzmann equation. The inset shows the quantitative comparison of voltages for half-maximal activation. Original figures from Husch et al. (2009a, b)

To encode odors, type I LNs, similar to uPNs, generate action potentials, while in type II LNs odors are mostly processed by graded changes in membrane potential. Accordingly, these neurons cannot trigger transmitter release by action potentials and are likely to release transmitters in a graded manner. The distinct intrinsic electrophysiological properties were reflected in the cell type-specific current profiles of the different AL neurons and in the biophysical properties of the expressed currents. So, for instance, the differences between spiking and nonspiking neurons are not only limited to the absence of action potentials and transient Na^+^ currents but are also clearly reflected in the biophysical properties of the voltage-activated potassium and calcium currents. This becomes particularly evident, for example, when comparing the functional parameters of voltage-activated Ca^2+^ currents (*I*_Ca_) between AL neurons (Fig. 4, bottom panels; Fig. 5).

### Voltage-activated Ca^2+^ currents and Ca^2+^ handling in AL neurons

In both type I and type II LNs, *I*_Ca_ starts to activate at membrane potentials around or below − 50 mV (Fig. 5b, d). These are relatively low activation thresholds compared with *I*_Ca_ in uPNs and in many other insect neurons (Byerly and Leung 1988; Saito and Wu 1991; Hayashi and Levine 1992; Laurent et al. 1993; Pearson et al. 1993; Wicher and Penzlin 1994, 1997; Schäfer et al. 1994; Kloppenburg 1999; Heidel and Pfluger 2006). Despite the relatively similar activation thresholds in both LN types, the half-maximal voltage for activation is significantly more hyperpolarized in type II LNs, compared with type I LNs (Fig. 4; Fig. 5b, d). Accordingly, the portion of *I*_Ca_ that can be activated at hyperpolarized membrane potentials is larger in type II LNs. This is consistent with the assumption that the synaptic release in type II LNs is regulated by graded changes in the membrane potential and not action potential-dependent.

While the *I*_*Ca*_ of both nonspiking LN types show important functional similarities, a more detailed analysis also reveals significant functional differences, of which the differences in voltage dependence for activation and in amplitude of the transient component are physiologically most relevant: The activation threshold of *I*_*Ca*_ is significantly lower and the amplitude of the transient component of the current is significantly larger in type IIa LNs compared with type IIb LNs (Husch et al. 2009b) (Fig. 5a, b). As a result, the total current inactivates faster in type IIa than in IIb LNs. The lower activation threshold and the relatively large transient component of *I*_Ca_ in type IIa LNs most likely contribute significantly to their active membrane properties, which include the generation of complex membrane depolarizations, including Ca^2+^-driven spikelets. Together, the low activation threshold and the large transient component could also mediate highly nonlinear membrane properties that amplify and sharpen excitatory postsynaptic potentials, even at hyperpolarized membrane potentials. In summary, this suggests that the strong active membrane properties of type IIa LNs correlate very strongly with the specific functional properties of their *I*_Ca_.

The different electrophysiological properties of the different AL neuron types, in particular their different *I*_Ca_, are also reflected in the significantly different cell type-specific Ca^2+^ handling properties: Nonspiking type II LNs, which do not possess Na^+^ currents, are strongly dependent on Ca^2+^ for membrane depolarization and exhibit large voltage-dependent Ca^2+^ currents. Compared with type I LNs and uPNs, both of which can generate Na^+^-driven action potentials and have significantly smaller Ca^2+^ currents, type II LNs have a higher Ca^2+^ binding ratio and Ca^2+^ extrusion rate (Pippow et al. 2009). It is plausible that these pronounced Ca^2+^ handling properties are crucial for handling the high Ca^2+^ load of the type II LNs to prevent a global uncontrolled (possibly toxic) increase of intracellular Ca^2+^.

### Transmitter diversity in LNs

**Fig. 6:**
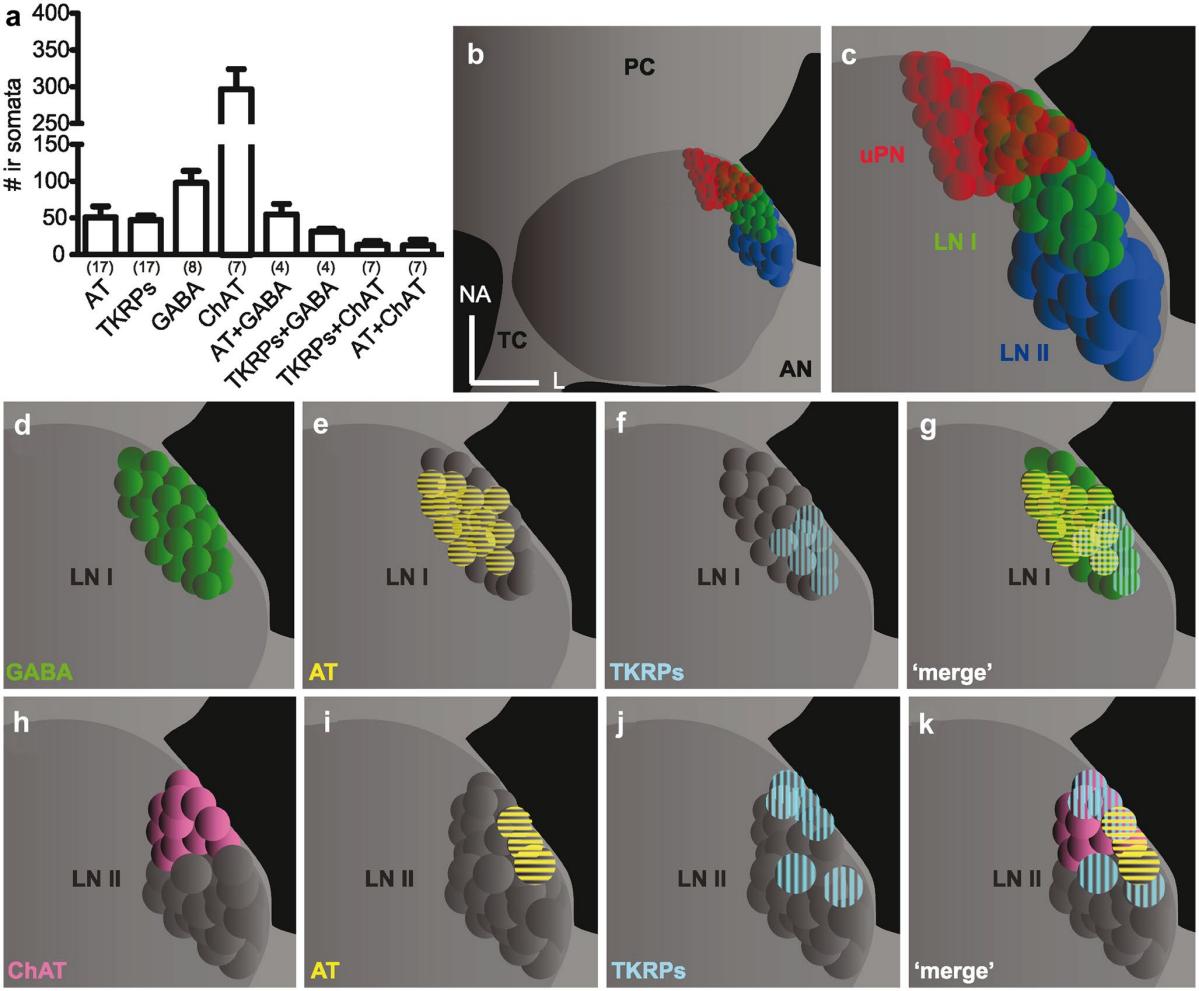
Overview of GABA-, ChAT-, AT-, and TKRP-like immunoreactive (-lir) somata in the ventrolateral somata group (VSG). **a** Numbers of immunoreactive somata for the respective transmitters and modulators. **b** Schematic illustration of a *P. americana* antennal lobe, including the VSG. **c** Higher magnification of the to demonstrate the somata positions of the different AL neuron types. **d–g** Position and immunoreactivity of type I LN somata. **h–k** Position and immunoreactivity of type II LN somata. GABA-lir somata are indicated in green, ChAT-lir somata are indicated in pink. Yellow bands indicate allatotropin-lir somata, cyan bands indicate TKRP-lir somata. AN antennal nerve, AT allatotropin, ChAT choline acetyltransferase, L lateral, LN I type I local interneurons, LN II type II local interneurons, NA n-anterior, PC protocerebrum, TKRPs tachykinin-related peptides, uPN uniglomerular projection neurons. Original figure from Fusca et al. (2015)

In line with their different morphological and physiological properties and the resulting differences in computational capacity and synaptic release mechanisms, LNs also exhibit different neurotransmitter and modulator profiles. Mass spectrometric and immunocytochemical studies assign a variety of neurotransmitters and peptides to identified LN types (Fig. 6).

For spiking type I LNs, GABA has been identified as the primary transmitter by immunohistochemical studies (Distler 1989; Husch et al. 2009a), and paired recordings confirmed fast GABA_A_- and slow GABA_B_ receptor-mediated inhibitory action of type I LNs onto uPNs and other type I LNs (Warren and Kloppenburg 2014). While apparently all spiking type I LNs exhibit GABA as their primary transmitter, they are biochemically not a homogeneous group. Early studies already showed dopamine-like immunoreactivity in a small subset of GABAergic LNs (Distler 1990b). More recent immunocytochemistry revealed expression of allatotropin (AT) and tachykinin-related peptides (TKRPs) in defined, overlapping subclusters in the type I LN somata cluster (Fusca et al. 2015). Furthermore, TKRPs, AT, and short neuropeptide F (sNPF) were found to be abundant in mass spectra of dissected type I LN somata clusters (Neupert et al. 2012).

In addition to the physiological and morphological diversity of nonspiking type II LNs, this cell type has an even higher biochemical diversity compared with type I LNs. A subset of type IIa LNs are immunoreactive for the ACh synthesizing enzyme choline acetyltransferase (ChAT), suggesting that they release ACh as their primary transmitter (Fusca et al. 2013). Additionally, single cell mass spectrometry confirmed the expression of ACh in a subset of type II LNs (Neupert et al. 2018). The primary transmitter of the remaining type II LNs however is still elusive. Nevertheless, a variety of neuropeptides that might act as neurotransmitters or -modulators could be detected in type II LNs. Mass spectrometry of tissue samples taken from the type II LN somata cluster showed highly variable peptidomes in different samples (Neupert et al. 2012). This variable neuropeptide composition was verified by single cell mass spectrometry of identified type II LNs, where multiple peptides were detected even in single cells (Neupert et al. 2018). Detected neuropeptides included TKRPs, AT, sNPF, extended FMRFamides, and allatostatin-C.

Accordingly, the classical transmitters GABA in spiking type I LNs and ACh in nonspiking type II LNs can be coexpressed with the neuropeptides AT, TKRPs, and sNPS and type I LNs can also coexpress dopamine (Distler 1990b; Fusca et al. 2015). A summary is shown in Figs. 4 and 6. While the functional significance of coexpression of classical transmitters with potential modulators has not been conclusively clarified, it is likely that these substances act as modulators on different time scales and ultimately contribute to synaptic plasticity. In fact, in connections between type I LNs and uPNs it can regularly be observed that a GABA_B_ receptor-mediated sustained inhibitory potential in the postsynaptic uPN is followed by a delayed slow depolarization. This depolarization even occurs after blocking the GABA induced hyperpolarization (Warren and Kloppenburg 2014), thus might hint to a cotransmitter or -modulator release.

The pronounced heterogeneity of olfactory LNs with respect to their biochemical profiles is consistent with studies in other insect species (Berg et al., 2007; Carlsson et al., 2010; Siju et al., 2013; reviewed in Homberg and Müller, 1999; Nässel and Homberg, 2006; Schachtner et al., 2005) as well as in the vertebrate olfactory bulb, where different transmitters and modulators have been assigned to morphologically and physiologically different local circuit neuron types (reviewed in Ennis et al., 2015). For example, coexpression of GABA and dopamine has also been described in periglomerular cells of the rat olfactory bulb. In this circuit, it is suggested that GABA is responsible for short-term inhibition, while long-term inhibition is accomplished by dopamine (Gall et al. 1987).

### Matching neuronal properties with their tasks

The summarized physiological, biophysical, and morphological studies on the single cell level of AL neurons in *P. americana* revealed cell type-specific functional phenotypes, which strongly implies differences in their computational properties and synaptic or modulatory output, and thus their functional role in the AL circuit.

In this regard, the main task of PNs, the coding, and transmission of the processed odor information over long distances from the AL to the protocerebrum are relatively well defined. This task requires the ability to generate fast action potentials in relatively high frequencies, which is not only reflected in the expression of transient Na^+^ currents but also in the expression of voltage- and Ca^2+^-activated K^+^ currents and their functional properties. The transient voltage-activated K^+^ current (*I*_*A*_) of uPNs, for example, has the lowest threshold, the fastest inactivation rate, and the highest current density of AL interneurons (Paeger et al. 2017). Together with the pronounced Ca^2+^-activated K^+^ currents (Bradler et al. 2016), this ensures rapid action potential repolarization and pronounced after-hyperpolarization, which in turn is a prerequisite for encoding information with high-frequency action potential firing.

The specific functional roles of the different LN types are less clear, especially the role of nonspiking type II LNs. The possibility that in addition and in parallel to spiking LNs, nonspiking LNs perform different tasks during olfactory processing has not been investigated in detail, although nonspiking LNs have been described in other insects (Tabuchi et al. 2015). Nevertheless, the different physiological properties of LN subtypes, which include different types of spiking and nonspiking neurons, have important consequences for their computational properties and the olfactory processing they perform.

The precise synaptic organization, which determines the synaptic input and output regions of the neuron, can only be shown by electron microscopic studies. In any case, some hypotheses about the synaptic input and output regions of the LNs can be formed from the distinct morphologies of these neurons. The spiking type I LNs, for example, expressed different branching patterns in different glomeruli, suggesting a polar organization with defined input and output regions (see also Distler and Boeckh, 1997b). The synaptic input from a defined receptive field (e.g., one or a few glomeruli) would be integrated at a local spike initiation zone into action potential firing that would spread to other innervated glomeruli and provide a defined set of glomeruli with synaptic input. In this model, glomeruli could interact independently of their distance: not only nearest-neighbor glomeruli could interact but also glomeruli that are distributed throughout the entire AL. The spiking, inhibitory, GABAergic type I LNs are a concrete example. In contrast, type II LNs have very similar branching patterns in all glomeruli, suggesting that they can receive synaptic input from all innervated glomeruli. However, during odor stimulation, synaptic input from olfactory receptor neurons will be typically restricted to certain glomeruli, in which graded postsynaptic potentials will be generated. In type IIa LNs with strong active membrane properties, the transient *I*_Ca_ component might boost and shape dynamic signals such as excitatory postsynaptic potentials. Such mechanisms might help to shape functional compartments, for example, for local integration, or they might help to activate nearby output synapses as suggested by Laurent et al. (1993). For nonspiking LNs with weakly active membrane properties, such as the type IIb LNs, interactions between glomeruli would be dependent on their (electrotonic) distance: potentials would spread only within the same glomerulus or to glomeruli that are electrotonically close to the stimulated glomerulus. These scenarios are hypothetical but seem to be testable with detailed structural, electrophysiological, and optophysiological studies together with structure and conductance-based modeling.

Taken together, the summarized data illustrate that the different AL neuron types show functionally important differences in physiology, structure, biochemistry, and computational capacity. Future studies have to find out how neurons with cell type–specific physiological characteristics, together with the expression of different transmitters and neuromodulators that can act on significantly different time scales, effectively mediate the processing of odor information in local AL circuits.

## Abbreviations

ACh: Acetylcholine
AL: Antennal lobe
ALT: Antennal lobe tract
AST-A: Allatostatin-A
AT: Allatotropin
ChAT: Choline acetyltransferase
iACT: Inner antenno-cerebral tract
*I*_*A*_: Voltage-activated transient K^+^ current
*I*_*Ca*_: Voltage-activated Ca^2^^+^ current
IPSP(s): Inhibitory postsynaptic potential(s)
lALT: Lateral antennal lobe tract
LH: Lateral horn
lir: Like immunoreactive
LN(s): Local interneuron(s)
L-PN: Large receptive field projection neuron
mALT: Medial antennal lobe tract
MG: Macroglomerulus
mlALT: Mediolateral antennal lobe tract
mPN(s): Multiglomerular projection neuron(s)
M-PN(s): Medium receptive field projection neuron(s)
oACT: Outer antenno-cerebral tract
OSN(s): Olfactory sensory neuron(s)
PN(s): Projection neuron(s)
sNPF: Short neuropeptide F
S-PN(s): Small receptive field projection neuron(s)
TKRPs: Tachykinin-related peptides
uPN(s): Uniglomerular projection neuron(s)
VSG: Ventrolateral somata group
